# Severe Spinal Cord Injury in Rats Induces Chronic Changes in the Spinal Cord and Cerebral Cortex Metabolism, Adjusted by Thiamine That Improves Locomotor Performance

**DOI:** 10.3389/fnmol.2021.620593

**Published:** 2021-03-29

**Authors:** Alexandra Boyko, Polina Tsepkova, Vasily Aleshin, Artem Artiukhov, Garik Mkrtchyan, Alexander Ksenofontov, Lyudmila Baratova, Sergey Ryabov, Anastasia Graf, Victoria Bunik

**Affiliations:** ^1^Faculty of Bioengineering and Bioinformatics, Lomonosov Moscow State University, Moscow, Russia; ^2^Belozersky Institute of Physico-Chemical Biology, Lomonosov Moscow State University, Moscow, Russia; ^3^Russian Cardiology Research-and-Production Complex, Ministry of Health of the Russian Federation, Moscow, Russia; ^4^Faculty of Biology, Lomonosov Moscow State University, Moscow, Russia; ^5^Faculty of Nano-, Bio-, Informational and Cognitive Technologies, Moscow Institute of Physics and Technology, Moscow, Russia; ^6^Department of Biological Chemistry, Sechenov University, Moscow, Russia

**Keywords:** amino acids metabolism, cerebral cortex, 2-oxoglutarate dehydrogenase, p53, phosphonate analog of 2-oxo acid, spinal cord injury, sirtuin 5, thiamine

## Abstract

Our study aims at developing knowledge-based strategies minimizing chronic changes in the brain after severe spinal cord injury (SCI). The SCI-induced long-term metabolic alterations and their reactivity to treatments shortly after the injury are characterized in rats. Eight weeks after severe SCI, significant mitochondrial lesions outside the injured area are demonstrated in the spinal cord and cerebral cortex. Among the six tested enzymes essential for the TCA cycle and amino acid metabolism, mitochondrial 2-oxoglutarate dehydrogenase complex (OGDHC) is the most affected one. SCI downregulates this complex by 90% in the spinal cord and 30% in the cerebral cortex. This is associated with the tissue-specific changes in other enzymes of the OGDHC network. Single administrations of a pro-activator (thiamine, or vitamin B1, 1.2 mmol/kg) or a synthetic pro-inhibitor (triethyl glutaryl phosphonate, TEGP, 0.02 mmol/kg) of OGDHC within 15–20 h after SCI are tested as protective strategies. The biochemical and physiological assessments 8 weeks after SCI reveal that thiamine, but not TEGP, alleviates the SCI-induced perturbations in the rat brain metabolism, accompanied by the decreased expression of (acetyl)p53, increased expression of sirtuin 5 and an 18% improvement in the locomotor recovery. Treatment of the non-operated rats with the OGDHC pro-inhibitor TEGP increases the p53 acetylation in the brain, approaching the brain metabolic profiles to those after SCI. Our data testify to an important contribution of the OGDHC regulation to the chronic consequences of SCI and their control by p53 and sirtuin 5.

## Introduction

Severe spinal cord injury (SCI) causes long-term consequences, similar to the traumatic brain injury (TBI). The consequences include cognitive decline and depression-like behavior, known to accompany neurodegenerative processes in CNS (Davidoff et al., 1992; Verweij et al., 1997; Robertson, 2004; Wu and Bogie, 2014; Wu et al., 2016; Jure and Labombarda, 2017). Neuroinflammation may link the CNS injuries to mental perturbations, as all these phenomena are involved with microglia activation (Chitnis and Weiner, 2017; Baufeld et al., 2018; Kinney et al., 2018; Pavlov et al., 2020) and dysregulation of cytokines (Allison and Ditor, 2015; Maldonado-Bouchard et al., 2016; do Espirito Santo et al., 2019; Wadhawan et al., 2019). Besides, inflammation is tightly associated with perturbed mitochondrial function, as is the immune response itself. A strong link between mitochondrial metabolism and immunity is clearly demonstrated by the fact that immune response is impaired upon inhibition of 2-oxoglutarate dehydrogenase multienzyme complex (OGDHC) (Diaz-Munoz et al., 2015), limiting the tricarboxylic acid (TCA) cycle flux (reviewed by Bunik and Fernie, 2009). Perturbed homeostasis of intracellular Ca^2+^, known to occur early after the CNS injuries and associated with increased levels of the reactive oxygen and nitrogen species (Verweij et al., 1997; Xiong et al., 1997; Scholpa and Schnellmann, 2017), may also be mediated by OGDHC, as Ca^2+^ ions activate OGDHC, stimulating the enzymatic production of both the energy intermediates and reactive oxygen species (reviewed in Bunik, 2019).

Several recent studies of animal models of TBI have addressed perturbations in mitochondrial metabolism in both acute (Mkrtchyan et al., 2018b; Pandya et al., 2019) and chronic (Hiebert et al., 2015) phases, ascribing a significant pathophysiological role to these changes. However, animal models of SCI are much less characterized in this regard, with most of the studies focusing on the spinal cord function. Moreover, perturbations of metabolism in the CNS area distant from the injured zone are usually left unattended. Our work aims to fill in the gaps by investigating the long-term alterations in central metabolism induced by SCI, with quantification of such alterations not only in the spinal cord, but also in cerebral cortex of the injured rats. We focus our primary attention on a key mitochondrial system, OGDHC, whose dysfunction is associated with a range of CNS diseases, including Alzheimer’s and Parkinson diseases, chronic neuroinflammation and brain injury (Mizuno et al., 1994; Gibson et al., 2005; Bunik et al., 2007; Mkrtchyan et al., 2018b). Arguably, the association originates from an essential role of OGDHC in the amino acid metabolism (Santos et al., 2006; Bunik and Fernie, 2009; Graf et al., 2009, 2020; Trofimova et al., 2012; Araujo et al., 2013) and immune response (Diaz-Munoz et al., 2015), i.e., the pathways known or implied to be changed in the chronic phases of CNS damage. In particular, changed metabolism of amino acids may underlie such important metabolic features of the damage as perturbed glutathione and nitric oxide (NO^⋅^) homeostasis, which are linked to cognitive perturbations (Aquilani et al., 2003; Gahm et al., 2006; Ploder et al., 2008; Vuille-Dit-Bille et al., 2012; Yi et al., 2016; Boyko et al., 2018; Xing et al., 2018). In our previous study of a rat model of severe SCI, a single dose of thiamine after SCI have attenuated long-term changes in the levels of the NO^⋅^- and glutathione-related amino acids in the brain cerebral cortex (Boyko et al., 2018). In a rat model of TBI, acute mitochondrial dysfunction due to perturbed function of OGDHC has been corrected by thiamine administration prior to the trauma (Mkrtchyan et al., 2018b). Positive action of a precursor of the coenzyme of the 2-oxo acid dehydrogenase complexes, thiamine (vitamin B1), or its pharmacological forms, in patients with neurodegenerative diseases has been reported since a long time (Blass et al., 1988; Costantini et al., 2013; Tapias et al., 2018; Gibson et al., 2020; Sambon et al., 2020). Thus, thiamine administration may help prevention of the chronic CNS damage after neurotrauma, which is considered as an essential goal of the current intensive care treatment of patients with CNS injuries (Stover et al., 2005; Vuille-Dit-Bille et al., 2012).

We have hypothesized that dysregulation of the brain OGDHC after severe SCI strongly contributes to chronic consequences of the trauma, with the pharmacological correction of the dysregulated metabolism by thiamine alleviating these consequences. In the current work employing the rat model of severe SCI, we demonstrate long-term effects of the injury on the function of OGDHC and affiliated metabolic enzymes in the CNS areas beyond the traumatized region, such as the total spinal cord and cerebral cortex. Using the amino acids profiling of the cerebral cortex as an integral marker of the SCI-induced metabolic perturbation in the brain, we show that a pleiotropic regulator thiamine, whose coenzyme form, thiamine diphosphate (ThDP), is required for the OGDHC function in particular, attenuates the changes in the known metabolic markers of the OGDHC downregulation in the brain. In contrast, administration of a pro-inhibitor of OGDHC mimics the SCI-induced changes in the brain amino acid profile. We also show that the observed metabolic changes are associated with the changed expression of the major metabolic regulators linked to OGDHC function, i.e., (acetyl)p53 and sirtuin 5.

## Materials and Methods

### Materials

If not specified otherwise, chemicals were obtained from Sigma-Aldrich (Helicon, Moscow, Russia). Thiamine^∗^HCl was from SERVA Electrophoresis GmbH. Deionized MQ-grade water was used to prepare solutions. Triethyl ester of glutaryl phosphonate (TEGP) was synthesized according to Artiukhov et al. (2020).

### Animal Husbandry

**FIGURE 1:**
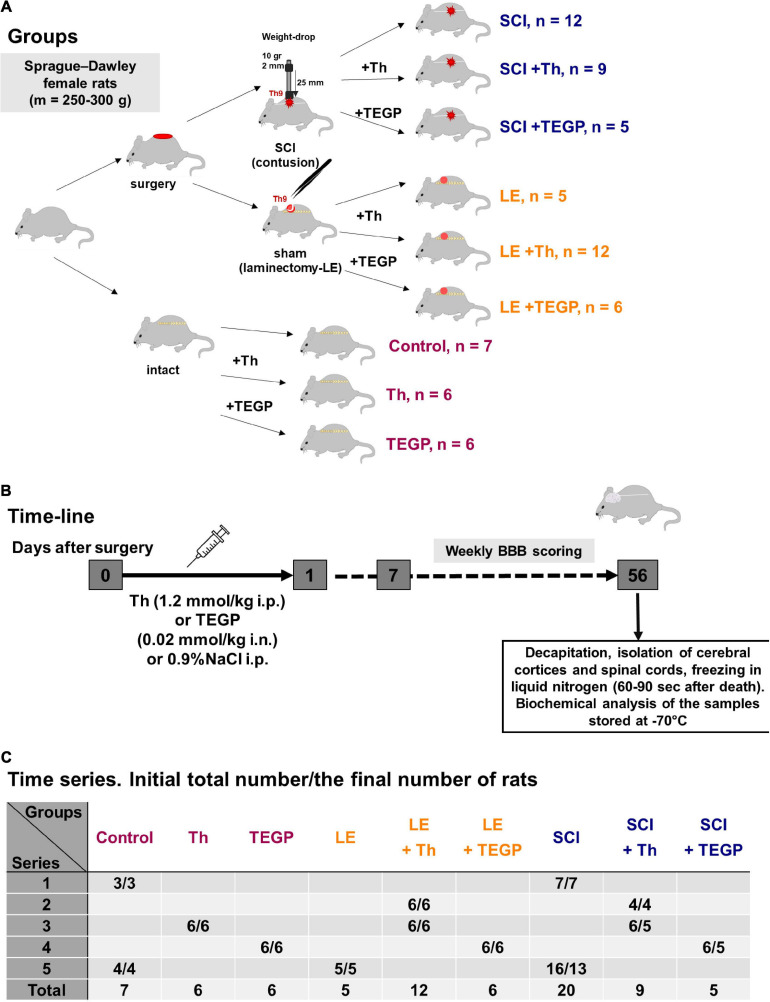
Experimental flow-chart. **(A)** The overview of experimental procedures for each animal group. **(B)** The time-line of the procedures. **(C)** The serial distribution of rats across the groups.

Manipulations with rats were carried out in accordance with the international recommendations of Good Laboratory Practice (GLP), methodical recommendations for laboratory animal care (Agricultural-Industrial Guidance Document 3.10.07.02-09), European Convention for the Protection of Vertebrate Animals Used for Experimental and Other Scientific Purposes, Strasbourg, 1986 ETS No. 123), as well as Guidelines for accommodation and care of animals, including species-specific provisions for laboratory rodents and rabbits developed by Rus-LASA (No. 33216-2014, 01.07.2016) and internal rules of Russian Cardiology Research-and-Production Complex. The experimental protocols were approved by Bioethics Committees of Russian Cardiology Research-and-Production Complex (Protocol No. 3, 23.03.2016). The study was not pre-registered. The minimum necessary size of the animal sample was estimated by *t*-test using a power of 80% and a level of significance of 0.05. Based on published data on the delayed effect of thiamine administration to rats on the ME activity in the cerebral cortex (averaged values ± SD 0.5 ± 0.1 vs. 0.8 ± 0.04 for the control and thiamine-treated groups) (Tsepkova et al., 2017). The minimal number of animals per group was estimated as three (Sample Size Calculation; Independent Sample *t*-test; two-tailed; STATISTICA version 10.0). From our past and current experiences, the loss of animals in the SCI model may be up to 20%, at least four rats per group were taken. Based on the results obtained in this first series of experiments, further animals were involved in the repeated series to better assess all the parameters estimated in this study. The rats were purchased from Nursery of laboratory animals, Institute of bioorganic chemistry (Pushchino, Russia). In total, 81 rats were involved in the study with 5 rats died during the post-surgical recovery period. The resulting 76 rats were distributed among the experimental groups as shown in Figure 1C. The study was exploratory, and no exclusion criteria were pre-determined.

The study was performed on female Sprague–Dawley rats. The adult rats of 12–13 weeks (weighting 230 ± 20 g) were exposed to LE or SCI, with their follow-up ended in 8 weeks, i.e., at the corresponding age of 20–21 weeks (weighting 290 ± 20 g in LE group and 265 ± 15 g in SCI group). The animals were kept in standard conditions with 12 h light and 12 h dark cycle in individual cages with free access to water and meal.

### Model of Severe SCI and Monitoring of Recovery

The SCI model and postsurgical care were described in details earlier (Ryabov et al., 2014; Boyko et al., 2018). Severe SCI was performed using the weight-drop method that allows maximal standardization of the injury level (Basso et al., 1996). Similar injury levels in different experimental animals subjected to SCI, were additionally supported by an acceptable range of standard error means (SEM) characterizing the time dependences of the animal locomotor recovery, measured as described below. The contusion injury at the T9 level was caused by a 10 g metal rod with a diameter of 2 mm, dropped from a height of 25 mm. In postoperative period, the animals were on a 30°C heating pad in individual cages, received anti-inflammatory drug gentamicin sulfate (1 mg/kg) during 7 days. At day 3 post-injury all the animals recovered from the operations and could eat, drink and move independently. Manual massage of the abdominal wall was carried out to empty the bladder until self-urination was restored. The white matter damage in our experiments was characterized in previous studies (Ryabov et al., 2014; Ryabov et al., 2020; Smirnov et al., 2020). Sham-operated animals were subjected to laminectomy (LE) at the T9 vertebra without affecting the dura matter. LE was associated with the formation of granuloma, affecting muscles, vessels and connective tissue at the site of the operation. Recovery of limb motor function was evaluated using the Basso, Beattie and Bresnahan rat locomotor scale (BBB) method from 0 (paralysis) to 21 points (normal walking) (Basso et al., 1995). Briefly, each rat was placed in an open field box (90 cm^2^ in area and 10 cm in surrounding walls) and videotaped for 4 min by digital camera. These video files (exemplified in Supplementary Materials) were viewed and evaluated blindly by an independent experimenter. Before injury, all animals underwent this test over 3 days. After injury, the testing was weekly over 8 weeks starting from day 7 post-operation. All procedures with animals were performed between 1 and 5 pm. Eight weeks after SCI rats were decapitated, and their cerebral cortices and spinal cords were taken for the analysis as described below. The overview of experimental procedures and all animal groups involved in the study is presented in Figure 1. The randomization procedure employed GraphPad QuickCalcs web-service^1^ as described earlier (Aleshin et al., 2020). The blinding was based on the fact that different experimenters performed separate steps of the study. That is, the persons who performed operations with animals, retrieved the tissues and labeled them by numbers, were not those who performed the biochemical assays of the tissues.

### Administration of the Small Molecule Regulators of OGDHC

Intraperitoneal injection of a neutralized water solution of 200 mg/ml thiamine hydrochloride (a dose of 1.2 mmol/kg) or intranasal administration of a water solution of 56 mg/ml trisodium salt of TEGP (a dose of 0.02 mmol/kg) were performed once in the morning following the operation, i.e., within 15–20 h after the operation. This experimental design imitated potential therapeutic intervention after the neurotrauma. Intranasal application was used as a non-invasive method providing an access to the CNS for different molecules that do not cross the blood-brain barrier (Dhuria et al., 2010). Control animals received the corresponding administration of physiological solution (0.9% NaCl).

### Rat Tissues, Their Extraction and Homogenization

After the rats were sacrificed, the spinal cord and brain were taken out and put on ice. From the spinal cord, the traumatized area (app. 4 mm) surrounded by the caudal and rostral regions (app. 7 mm each) was taken for the analyses (Smirnov et al., 2020). Taking into account the earlier studies on the spinal cord changes in the traumatized area, the contribution of the scar tissue was estimated to be within 20% of the total spinal cord tissue taken for analysis (Ryabov et al., 2014; Ryabov et al., 2020; Smirnov et al., 2020). The cerebral hemispheres (further called the cortex) were separated from other brain parts. The rapidly (≤120 s) prepared tissue samples were frozen in liquid nitrogen and stored at –70°C before biochemical analyses. To assay the enzymatic activities, the whole sample of the extracted spinal cord and a half of the cortex tissue were homogenized according to the previously published protocol (Tsepkova et al., 2017). Homogenization buffer contained 50 mM MOPS pH 7.0, 2.7 mM EDTA, 20% glycerol and protease inhibitors. For metabolic profiling, another half of the brain cortex was extracted with methanol and acetic acid according to the published procedure (Ksenofontov et al., 2017; Boyko et al., 2018).

### Enzymatic Assays

In view of the high physiological significance of the TCA cycle and affiliated amino acid metabolism in CNS, we focused on the selected enzymes of these metabolic pathways in SCI. The selection included the interacting and most regulated enzymes of the glutamate node, such as OGDHC, glutamate dehydrogenase (GDH) and glutamine synthetase (GS), and the enzymes of the pyruvate/malate metabolism, such as PDHC, NADP^+^-dependent malic enzyme (ME) and malate dehydrogenases (MDH), linked to the glutamate node through the TCA cycle and transamination reactions (Supplementary Figure S1). The activities of enzymes were measured after solubilisation of the mitochondria in the brain homogenate by ultrasound and detergents in 0.2 ml of a reaction medium on a Tecan Sunrise microplate reader (Austria) as described earlier (Tsepkova et al., 2017). In view of the limited volume of the homogenates, the multienzyme assay scheme ensured that each enzymatic activity was determined in at least four animals of a group, using at least three technical replicates for each sample. Activities of enzymes are expressed in μmol of a product generated per min per g of the tissue fresh weight (FW). The activity measurements were done at saturating concentrations of all the substrates and cofactors. The maximal reaction rate of an enzyme or enzymatic complex was thus estimated, corresponding to the expression of a functional enzyme or its multienzyme complex in the tissue homogenate.

### Quantification of the GDH Acetylation and Expression by Mass-Spectrometry

The quantification employed nano-LC-MS/MS analysis on a hybrid dual-pressure linear ion trap/orbitrap mass spectrometer (LTQ Orbitrap Velos Pro, Thermo Scientific, San Jose, United States) equipped with an U3000 nano-flow HPLC (Thermo Scientific, San Jose, CA) as described previously (Aleshin et al., 2020). Briefly, the samples were obtained after the in-gel trypsinolysis of the gel areas corresponding to GDH. The level of K503 acetylation was normalized to the same non-acetylated peptide ISGASE**K**DIVHSGLAYTMER. The normalization of the acetyl-K415-comprising peptide IIAEGANGPTTPEAD**K**IFLER employed the sum of the two non-acetylatable and well-detectable peptides of GDH, i.e., DDGSWEVIEGYR and DSNYHLLMSVQESLER. These peptides were also used to estimate the GDH expression. The comparison of the GDH protein level between different samples employed normalization to the total ion current during the mass-spectrometric analysis of the samples (Aleshin et al., 2020).

### Quantification of the Sirtuin 5 and p53 Levels and p53-K380 Acetylation by Western-Blotting

The levels of sirtuin 5, p53 and acetylated p53 proteins were estimated using primary antibodies from Cell Signaling Technology (#8782; Leiden, The Netherlands) and Thermo Fisher Scientific (CA, United States)—#PA5-17287 designed for mouse K379 acetylated p53 (equal to human K382 or rat K380 residue) and #MA5-12453 for total p53. The primary antibodies for sirtuin 5, total p53 and acetylated p53 were used in 1:1,000, 1:350 and 1:700 dilutions, respectively, with the appropriate secondary HRP-conjugated antibodies. The relative quantification of chemiluminescence was performed in ChemiDoc^TM^ Imager (Bio-Rad, California, United States) and Image Lab software version 6.0.1 (Bio-Rad, California, United States). As single proteins, such as beta-actin or GAPDH, traditionally used for the loading control due to their abundance, were later shown to undergo changes under different conditions (Lowe et al., 2000; Dittmer and Dittmer, 2006; Guo et al., 2010; Hu et al., 2016), normalization of the protein levels to the total protein in the corresponding gel lanes was performed, using the fluorescent quantification of the total protein in each lane with 2,2,2-trichloroethanol, according to the published procedure (Ladner et al., 2004). The band intensities from different membranes were compared across all the membranes after the normalization on different levels of the samples repeated on independent membranes.

### Metabolic Profiling of the Rat Brain Extracts

Amino acids and related compounds were quantified in extracts of cerebral cortices according to Ksenofontov et al. (2017), Boyko et al. (2018) using an amino acid analyzer L-8800 (Hitachi Ltd., Japan). HPLC employed a gradient of Li-citrate buffers, followed by modification of amino acids with the ninhydrin reagent (Wako Pure Chemical Industries; P/N 298-69601). Glutathione disulphide (GSSG) was quantified using fluorescence of its product with o-phtalic aldehyde according to the method (Hissin and Hilf, 1976) optimized as in Senft et al. (2000). Tryptophan levels in the brain extracts were determined as described in Denckla and Dewey (1967) with modifications according to Bloxam and Warren (1974), using the tryptophan conversion into fluorescent norharman. The fluorescent signal was obtained at λ ex/λ em of 365 nm/460 nm.

### Statistical Analysis

All data were analysed using GraphPad Prism 7.0 software (GraphPad Software, Inc., United States) or R Studio^2^ and calculated as mean ± SEM. On the figures, biochemical parameters are presented as boxes and whiskers, showing quantiles of each sample distribution. Comparisons of SCI and LE groups of animals were performed with unpaired Mann-Whitney’s test. When the normality of the data distribution was confirmed using Shapiro-Wilk test, *t-*test was employed for the comparison of the two groups. For comparison of more than two groups, two-way analysis of variance (ANOVA) and *post hoc* Tukey’s test were used. To estimate the statistical difference between the time-dependent locomotor recovery curves of rats after SCI without or with treatment, the extra sum-of-squares *F*-test was used. Correlations between the pairs of parameters were characterized by the Spearman’s correlation coefficients and *p-*value of the correlation. The ROUT test for outliers (Motulsky and Brown, 2006) was applied. In all cases, two-tailed *p*-values ≤ 0.05 and 0.05 < *p* ≤ 0.10 were considered to indicate statistical significance of differences and trends, respectively. The *p*-values are marked by asterisk(s) in the figures according to the following code: ^∗^ ≤ 0.05, ^∗∗^ ≤ 0.01, ^∗∗∗^ ≤ 0.001, ^****^ < 0.0001.

## Results

### Chronic Changes in the Activities of Metabolic Enzymes in Spinal Cord and Cerebral Cortex After SCI

**FIGURE 2:**
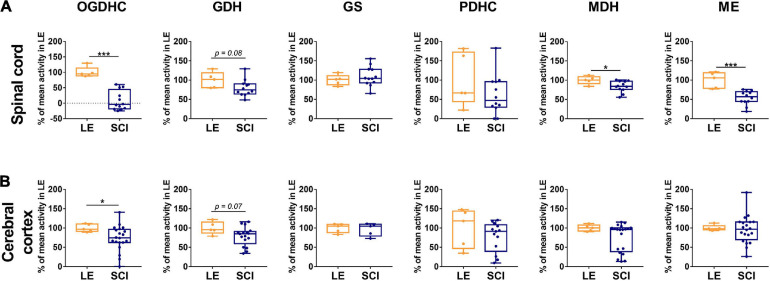
The long-term effect of severe spinal cord injury (SCI) on the enzymatic activities in spinal cord **(A)** or cerebral cortex **(B)**, compared to those of the rats subjected to laminectomy (LE). The assayed activities are indicated in the upper part of the figure, the enzymes are indicated as in Supplementary Figure S1. Data are expressed as percentage of the activity level in LE group. Statistical significance of differences between groups is estimated using Mann-Whitney’s test. Different *p*-values are shown by asterisks as described in Methods under Statistical Analysis. Number of animals analyzed in each group: LE, *n* = 5; SCI, *n* ≥ 5.

The activities of selected enzymes of central metabolism (Supplementary Figure S1) in the spinal cord and cerebral cortex have been compared between the sham-operated (LE) and SCI animal groups (Figure 2). As shown in Figure 2A, 8 weeks after SCI, activities of the assayed enzymes in the spinal cord do not change to a similar degree. The disproportionality of changes in different enzymatic activities underlines that these changes cannot be attributed to diminished protein content due to existence of the cavity and surrounding scar tissue. From the assayed enzymes, OGDHC in the spinal cord of the animals with severe SCI shows the highest degree of the downregulation, compared to LE group (Figure 2A). While the OGDHC activity of the spinal cord in LE animals is 0.41 ± 0.03 μmol/min per g of FW, the residual level of the OGDHC activity in the spinal cord of SCI animals (0.04 ± 0.04 μmol/min per g of FW) is comparable to the background values (app 10% of the initial activity). This is accompanied by a less significant (p = 0.08) 20% decrease of GDH (Figure 2A), whose activity is characterized by the mean values 2.30 ± 0.21 and 1.83 ± 0.14 μmol/min per g of FW in the LE and SCI animals, correspondingly. The two enzymes are also downregulated in cerebral cortex (Figure 2B), where SCI decreases the OGDHC activity by about 30%, from 1.06 ± 0.05 μmol/min per g of FW in the LE group to 0.78 ± 0.08 μmol/min per g of FW in SCI animals. The brain cortex GDH shows a 20% decrease (*p* = 0.07), similar to that observed in the spinal cord, with the activity mean values changing from 1.34 ± 0.10 μmol/min per g of FW in the LE group to 1.08 ± 0.08 μmol/min per g of FW in the SCI group. Apart from the higher level of OGDHC downregulation, the difference between the spinal cord and cerebral cortex of the SCI vs. LE rats is manifested in the activities of the malate-dependent enzymes. ME and MDH are decreased in the spinal cord, but remain unaffected in the cerebral cortex (Figure 2).

A decrease in the TCA cycle flux due to the downregulation of OGDHC (Figure 2B) is obvious from elevated levels of alanine, serine, proline and aspartate (Table 1). The Fischer ratio, known to be of prognostic value upon TBI, inflammation and sepsis (Chiarla et al., 2011; Vuille-Dit-Bille et al., 2012), is increased (Table 1). The level of glutathione does not significantly change as a chronic consequence of SCI, compared to LE, although the level of glutathione disulfide (GSSG) tends to rise. Overall, the quantification of free amino acids and related compounds in the extracts of cerebral cortex of the animals of the LE and SCI groups reveal that even the less expressed changes of enzymatic activities in the cortex, compared to the spinal cord, are accompanied by a significant perturbation of the brain metabolome.

**TABLE 1:** Metabolic profiling of free amino acids and related compounds in the brain cortex of the rats subjected to laminectomy (LE) or severe spinal cord injury (SCI), non-treated or after administration of thiamine or TEGP.

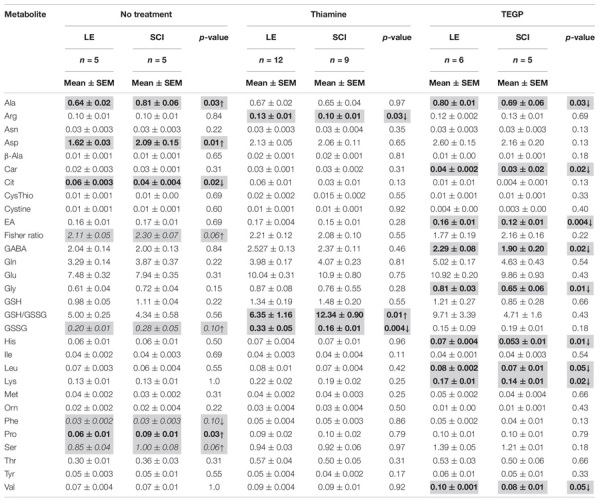

*The levels of metabolites are presented in μmol per g of FW as mean ± standard error of mean (SEM). Statistical significances of the differences between the pair of groups are estimated with unpaired Mann-Whitney’s test. Significant differences (p ≤ 0.05, in bold) and trends (0.05 < p ≤ 0.1, in italic) are highlighted in gray. Arrows near the p-values specify the directions of the changes. Fisher ratio is the sum of isoleucine, leucine and valine levels, divided by the sum of tyrosine and phenylalanine levels (Soeters and Fischer, 1976). Proteinogenic amino acids are named by their standard three-letter abbreviations. β-Ala (β-alanine); Car, carnosine; Cit, citrulline; CysThio, cystathionine; GABA, γ-aminobutyric acid; GSH, glutathione (reduced form); GSSG, glutathione disulfide; EA, ethanolamine; Orn, ornithine. n – Number or animals in a group.*

Thus, there are long-term functional rearrangements of central metabolism in CNS of animals with neurotrauma (SCI group), compared to LE group. Eight weeks after SCI, OGDHC is significantly downregulated not only in the area of the spinal cord, surrounding the trauma at T9 level in the rostral and caudal directions, but also in the cerebral cortex, that has not been exposed to mechanic trauma (Figure 2). The downregulation-imposed limitations in the metabolic flux through the TCA cycle are expressed in the perturbed homeostasis of amino acids in the cortex of SCI animals, compared to LE animals (Table 1).

### Regulation of the Brain and Spinal Cord Metabolism by Thiamine Administration After SCI

**FIGURE 3:**
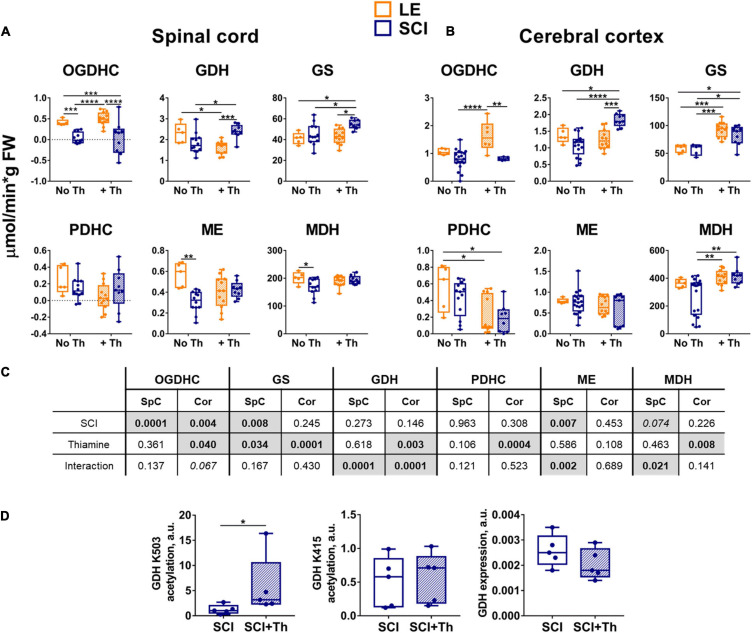
Reactivities of the OGDHC and affiliated enzymes to the post-operative administration of the OGDHC pro-activator thiamine in the LE and SCI rats. **(A)** The enzymatic activities in the spinal cord (SpC). **(B)** The enzymatic activities in the cerebral cortex (Cor). **(C)** Significances (*p* ≤ 0.05, in bold) and trends (0.05 < *p* ≤ 0.1, in italic) of the SCI and thiamine factors and their interactions, according to the two-way ANOVA of the data in **(A,B)**, are highlighted in gray. **(D)** Acetylation of the GDH residues K504 and K415 and the GDH expression: Acetylation level of K503 is normalized to the same peptide comprising the deacetylated K503; acetylation level of K415 is normalized to the sum of the two reference GDH peptides, which are also used for the estimation of the GDH expression; significance of differences between the two groups is estimated using Mann-Whitney’s test. Thiamine is administered intraperitoneally at a dose of 1.2 mmol/kg within 24 h after operation. On the graphs, different *p*-values are shown by asterisks as described in Methods under Statistical Analysis. Number of animals in each group: LE, n = 5; SCI, *n* ≥ 5; LE + Th, *n* ≥ 6; SCI + Th, *n* ≥ 5.

A decrease in the OGDHC activity is known to be addressed by increased thiamine influx into the affected neurons and brain (Mkrtchyan et al., 2016). Moreover, the non-coenzyme binding of thiamine or its derivatives also regulates other enzymes of the considered pathways (Mkrtchyan et al., 2015; Tsepkova et al., 2017; Aleshin et al., 2019; Aleshin et al., 2020; Mezhenska et al., 2020). Complex effects of the thiamine administration on the SCI-perturbed pathways (Figure 3) are in good accord with these previous studies. Comparison of the thiamine action in SCI and LE animals shows that the thiamine effects are often non-additive to the effect of SCI (Figure 3). The complexity is manifested by the results of the ANOVA analysis regarding not only the statistical significance, but also the interaction of the studied factors, i.e., SCI and thiamine administration (Figure 3C). In the cerebral cortex, the factor of thiamine treatment shows statistical significance for almost all the enzymatic activities tested (except for ME changing at the level of a trend, *p* = 0.1). Besides, the thiamine treatment interacts with the factor of trauma in case of the GDH activity (Figure 3C). The interaction is manifested in the opposite effects of the thiamine administration on the GDH activity in the LE and SCI groups (Figure 3B). In the spinal cord, the factor of thiamine administration is significant for GS activity and strongly interacts with the factor of trauma, i.e., has very different actions in the LE and SCI groups, when the GDH, ME and MDH activities are considered (Figure 3C).

Thiamine administration does not significantly change OGDHC activity in the spinal cord of either LE or SCI groups (Figure 3A). However, in the cerebral cortex it increases the OGDHC downregulation in SCI vs. LE animals (Figure 3B). Simultaneously, thiamine causes the tissue-specific effects on the affiliated enzymes of the metabolic nodes of glutamate and malate (Figure 3). In the spinal cord of the non-treated animals, OGDHC, ME and MDH are downregulated in SCI vs. LE rats, but after thiamine administration the downregulation of ME and MDH in SCI vs. LE rats is not observed; instead, GDH and GS are upregulated (Figure 3A). In the cerebral cortex, administration of thiamine also upregulates the GDH activity in SCI vs. LE animals (Figure 3B). In view of the recently characterized thiamine effects on the GDH acetylation (Aleshin et al., 2020), we assessed the thiamine involvement in the regulatory acetylation of GDH in SCI animals. Figure 3D shows that in the brain of SCI animals a statistically significant increase in the relative acetylation level of the GDH residue K503 is induced by the thiamine administration. The treatment does not change either the acetylation of another GDH residue, K415, nor the total GDH level (Figure 3D). As the acetylation of K503 in the inhibitory GTP site is known to increase the activity of GDH (Aleshin et al., 2020), the thiamine-induced elevation in acetylation of K503 (Figure 3D) represents the molecular mechanism increasing GDH activity in SCI animals (Figure 3B). Thus, the non-coenzyme action of thiamine in regulatory acetylation of GDH is involved in the pleiotropic effects of thiamine on the chronic metabolic changes in the rat brain after SCI.

The complex action of thiamine on the enzymes functioning in the cerebral cortex (Figures 3D,C) counteracts the SCI-induced changes in the brain metabolic profiles, compared to LE. In fact, after the thiamine administration, multiple differences in the levels of free amino acids between the non-treated SCI and LE animals are no more observed (Table 1). In particular, the thiamine treatment abrogates the negative effects of SCI on the Fischer ratio and GSSG (Table 1). Thus, the thiamine treatment normalizes the SCI-induced perturbations in the brain metabolic profile (Table 1).

### Regulation of the Brain and Spinal Cord Metabolism After SCI by a Specific Inhibitor of OGDHC

**FIGURE 4:**
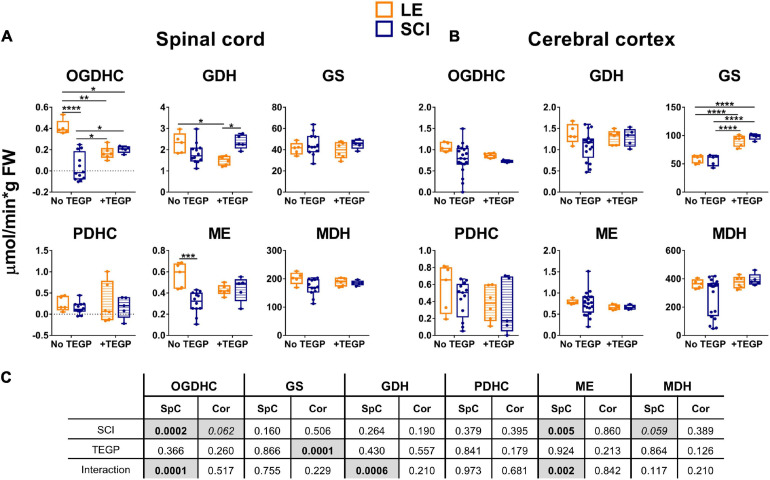
Reactivities of OGDHC and affiliated enzymes to the post-operative administration of the OGDHC pro-inhibitor TEGP in the SCI or LE rats. **(A)** The enzymatic activities in the spinal cord (SpC); **(B)** The enzymatic activities in the cerebral cortex (Cor); **(C)** Significances (*p* ≤ 0.05, in bold) and trends (0.05 < *p* ≤ 0.1, in italic) of the SCI and TEGP factors and their interactions, according to the two-way ANOVA of the data in **(A,B)**, are highlighted in gray. TEGP is administered intranasally at a dose of 0.02 mmol/kg within 24 h after operation. On the graphs, different *p*-values are shown by asterisks as described in Methods under Statistical Analysis. Number of animals in each group: LE, *n* = 5; SCI, *n* ≥ 5; LE + TEGP, *n* = 6; SCI + TEGP, *n* = 5.

A membrane-permeable (triethylated) precursor (TEGP) of the OGDHC inhibitor glutaryl phosphonate (GP, Supplementary Figure S2A), which is formed from TEGP by intracellular esterases, has been used for the OGDHC-directed inhibition. Compared to the previously studied OGDHC inhibitor of this class, succinyl phosphonate (SP, Supplementary Figure S2A), glutaryl phosphonate is a stronger inhibitor of the brain OGDHC (Supplementary Figure S2B). At the same time, selectivity of the glutaryl phosphonate action regarding OGDHC vs. other enzymes, including those studied in this work, is very high and similar to that of succinyl phosphonate (Artiukhov et al., 2020). Thus, unlike the pleiotropic action of thiamine having both the coenzyme and non-coenzyme protein targets, TEGP targets *in vivo* are limited to OGDHC. Shortly after the trauma, the tight but reversible binding of the TEGP-generated glutaryl phosphonate at the 2-oxoglutarate binding site may be expected to protect the enzyme from the irreversible inactivation caused by the reactive oxygen and nitrogen species rising at the site of SCI damage. However, based on pharmacokinetics of other water-soluble xenobiotics, TEGP is supposed to be excreted from the organism long before our tissue extraction, which has been done 8 weeks after the trauma. Yet the biochemical analyses of the tissues show that TEGP treatment has the delayed metabolic effects. In the spinal cord, it strongly reduces the OGDHC activity in the LE animals, increasing the activity in the SCI animals (Figure 4A). The interaction of the effects of the TEGP administration with those of the operations is also revealed for the GDH and ME activities in the spinal cord (Figures 4A,C). In the cortex, the TEGP effect on the functional expression of the enzymes is limited to a significant upregulation of GS both in the SCI and LE animals (Figure 4B). Thus, the interaction of TEGP with the SCI-induced changes is pronounced in the spinal cord, but in the cerebral cortex, where TEGP acts similarly in the LE and SCI animals (Figure 4C).

A specific effector of OGDHC, TEGP does not affect PDHC (Artiukhov et al., 2020). Accordingly, in case of the TEGP treatment, there are no concerted changes in the metabolic enzymes of both the 2-oxoglutarate/glutamate and pyruvate/malate nodes (Figure 4), observed after the thiamine administration (Figure 3). This difference between the TEGP- and thiamine-dependent metabolic regulation is manifested in the corresponding metabolome profiles (Table 1). While thiamine administration normalizes the levels of the SCI-affected amino acids, in the TEGP-treated SCI animals the levels of many amino acids decrease compared to those in LE animals (Table 1).

### Effects of the Thiamine or TEGP Administration on the Locomotor Recovery After SCI

**FIGURE 5:**
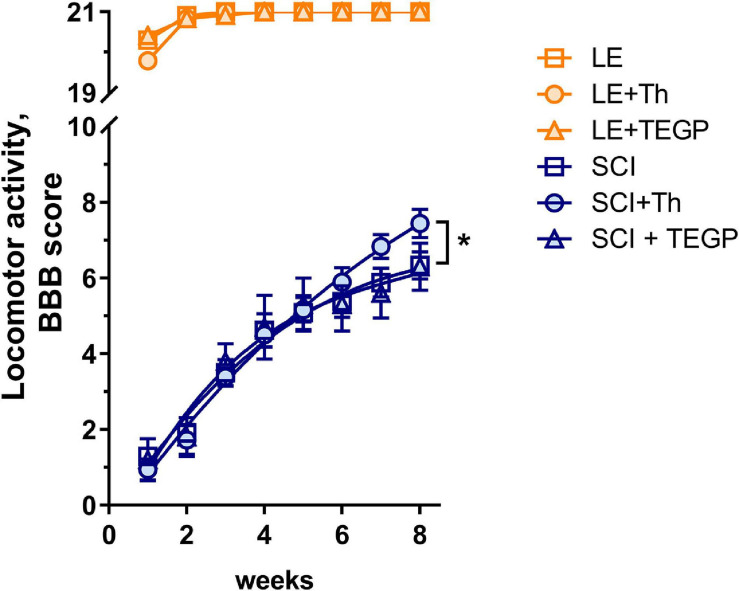
Locomotor recovery during 8 weeks after SCI or LE in the non-treated rats and those with the post-operative administration of thiamine (Th) or TEGP. The recovery of locomotion is scored from 0 to 21 using the BBB scale (Basso et al., 1995). Number of animals in each group: LE, *n* = 5, LE + Th, *n* = 12, LE + TEGP, *n* = 6, SCI, *n* = 12, SCI + Th, *n* = 9, SCI + TEGP, *n* = 5. Data is fitted with the third order polynomial equation. Significance of the differences between the approximation curves is estimated by the extra sum-of-squares *F*-test. *P*-value < 0.05 is shown by asterisk.

To find whether/how the observed effects of thiamine and TEGP on the central metabolic enzymes in CNS affect the locomotor recovery of injured animals, we have studied the hind limbs function of the rats during 8 weeks after the trauma, which represents the functional outcome of the SCI-induced white matter damage (Basso et al., 1995). The recovery is scored using the Basso, Bresnahan, Beattie (BBB) scale. As seen from Figure 5, all sham-operated rats recover their locomotor function up to the maximum score of 21 during the first 2 weeks following the operation, with no significant influence on the score by either thiamine or TEGP. The rats exposed to severe SCI do not reach the original score even 8 weeks after the trauma (Figure 5). However, according to sum-of-squares F-test, the locomotor recovery curves for the animals treated and non-treated with thiamine, differ significantly, revealing a positive effect of the thiamine administration on the locomotor performance of the animals after about 6 weeks of the recovery. At the end of the monitoring, the thiamine-treated SCI rats exhibit an 18% improvement in their locomotor performance, compared to the SCI rats which did not obtain thiamine (Figure 5). Thus, the thiamine administration within 15–20 h after SCI improves the chronic consequences of the neurotrauma. In contrast, the curve of the locomotor recovery of rats with the post-SCI administration of TEGP does not differ from the curve for the non-treated SCI animals (Figure 5). Thus, the concerted action of thiamine on the enzymes of both the glutamate and pyruvate/malate metabolism (Figure 3), normalizing the brain amino acid profile (Table 1), improves locomotor performance. In contrast, the TEGP action (Figure 4) neither normalizes the brain metabolome (Table 1), nor improves the locomotor performance (Figure 5).

To identify metabolites and pathways correlating with the locomotor recovery after SCI, we have analyzed the relations between the levels of cerebral cortex metabolites or enzymes and BBB scores in all the SCI-subjected rats. As shown in Table 2, β-alanine, cystathionine, glutamate, lysine and phenylalanine exhibit moderate (R∼0.5–0.6) statistically significant (*p* ≤ 0.05) positive correlations with the BBB scores, whereas the moderate (R∼0.4) correlations with GABA, leucine and isoleucine are less significant (*p* < 0.10). From the tested enzymes, PDHC shows a strong statistically significant (*p* < 0.01) negative correlation with the BBB score, whereas GDH exhibits a moderate positive correlation with BBB, although of a lower significance (*p* = 0.07) (Table 2).

**TABLE 2:** Correlations between the level of locomotor recovery of rats 8 weeks after SCI and metabolites in the cerebral cortex.

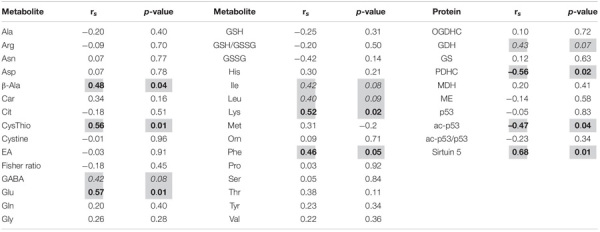

*The Spearman correlations are estimated in all the SCI-subjected rats, non-treated and treated with thiamine or TEGP (n = 19). The significant (p ≤ 0.05) correlations of metabolites with BBB scores are shown in bold, the trends (0.05 < p ≤ 0.1) are shown in italics. Metabolites are abbreviated as in Table 1.*

### The Long-Term Changes in Metabolism of Cerebral Cortex by OGDHC Regulators Depend on the (Patho)physiological States of the Animals

In animal models, comparison of biochemical parameters is done across different (patho)physiological states and treatments, which is needed to study molecular mechanisms of the pathology. However, human studies usually employ comparison of the patients with neurotrauma to control subjects (Ploder et al., 2008; Vuille-Dit-Bille et al., 2012; Yi et al., 2016). Hence, to understand the action of the studied treatments in different (patho)physiological states, in addition to the comparison of the LE and SCI animals, we studied the influence of thiamine and TEGP also in the control non-operated animals.

**FIGURE 6:**
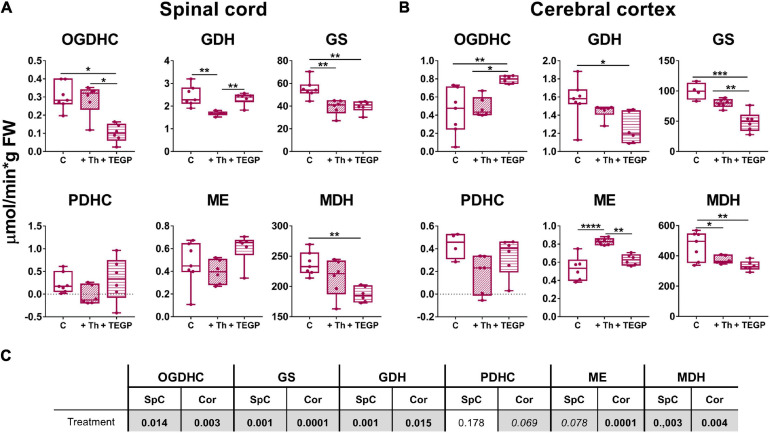
Reactivities of OGDHC and affiliated enzymes to the thiamine or TEGP administration in the non-operated rats. **(A)** The enzymatic activities in the spinal cord (SpC), **(B)** The enzymatic activities in the cerebral cortex (Cor). **(C)** Significances (*p* ≤ 0.05, in bold) and trends (0.05 < *p* ≤ 0.1, in italic) of the treatment factor, according to one-way ANOVA of the data in **(A,B)**, are highlighted in gray. Thiamine (Th, 1.2 mmol/kg intraperitoneally) or TEGP (0.02 mmol/kg intranasally) are administered to non-operated (control, **C**) rats. On the graphs, different *p*-values are shown by asterisks as described in Methods under Statistical Analysis. Number of animals in each group: Ctrl, *n* ≥ 4; TEGP, *n* = 6; Th, *n* = 6.

As shown in Figures 3, 4, OGDHC regulators affect the enzymes differently in the LE and SCI animals. This is further confirmed by the changes in the assayed enzymes due to the thiamine or TEGP administration to the control rats (Figure 6). Administration of the OGDHC activator thiamine does not change the maximal activity of OGDHC, assayed when the coenzyme and substrates are saturating, in either spinal cord or cortex homogenates (Figure 6). However, the tissue-specific reorganization of the enzymes affiliated with OGDHC and/or TCA cycle is observed after the thiamine treatment of control animals: Compared to the non-treated controls, the spinal cord activities of GDH and GS decrease, whereas the cerebral cortex activities of ME and MDH decrease and increase, correspondingly. The findings suggest that the thiamine administration affects the thiamine-dependent substrate flux through OGDHC *in vivo* even if the functional expression of the complex, i.e., its maximal activity in the brain homogenates, assayed under the substrate saturation, remains unaffected (Figure 6).

Dependence of the effects of the OGDHC regulators on the animal state may be caused by different levels of the studied enzymatic activities in these states. For instance, in the control rats, TEGP significantly increases the OGDHC activity in the cortex (Figure 6B), while no such action of TEGP is observed in the LE or SCI animals (Figure 4B). However, in the latter states, compared to the control cortices, the OGDHC is already upregulated to the values induced by the TEGP treatment of the control rats (≈ 1 μmol/min per g FW). The opposite situation is observed with GS: TEGP decreases GS activity in the control rats (Figure 6) to the levels observed in the LE and SCI animals (≈ 50 μmol/min per g FW, Figure 4B), but upregulates the enzyme in the LE and SCI animals (Figure 4) to the levels, inherent in the control cortices (∼100 μmol/min per g FW, Figure 6). Thus, TEGP action on the enzymes in the control animals resembles the actions of LE and SCI.

Similar to the LE- or SCI-exposed animals, the control animals also show the tissue-specific responses of the tested enzymes to the OGDHC inhibition. TEGP downregulates OGDHC in the spinal cord (Figure 6A), but induces a strong upregulation of the enzyme in the cerebral cortex (Figure 6B). The tissue-specific regulation is inherent also in the OGDHC interaction partner GDH. In the control rats, the TEGP treatment downregulates GDH in the cortex (Figure 6B), but does not change GDH in the spinal cord (Figure 6A). However, GS and MDH are reduced after the TEGP treatment of the control animals both in the spinal cord and cerebral cortex (Figure 6). Thus, inhibition of OGDHC *in vivo* causes tissue-specific adjustments in the metabolically linked enzymes.

Overall, the studied enzymatic network is more responsive to either of the OGDHC regulators in the control animals, than in the SCI-exposed animals. In particular, TEGP significantly affects MDH in the control rats (Figure 6), which is not observed in the rats after SCI (Figure 4). This finding testifies to a higher homeostatic response to metabolic regulators in the control animals, compared to those injured. As a result, changes in the functions of the OGDHC-affiliated enzymes due to the action of the OGDHC regulators depend on the animal state, interacting with the effects of sham operation and SCI (Figures 3, 4, 6).

**FIGURE 7:**
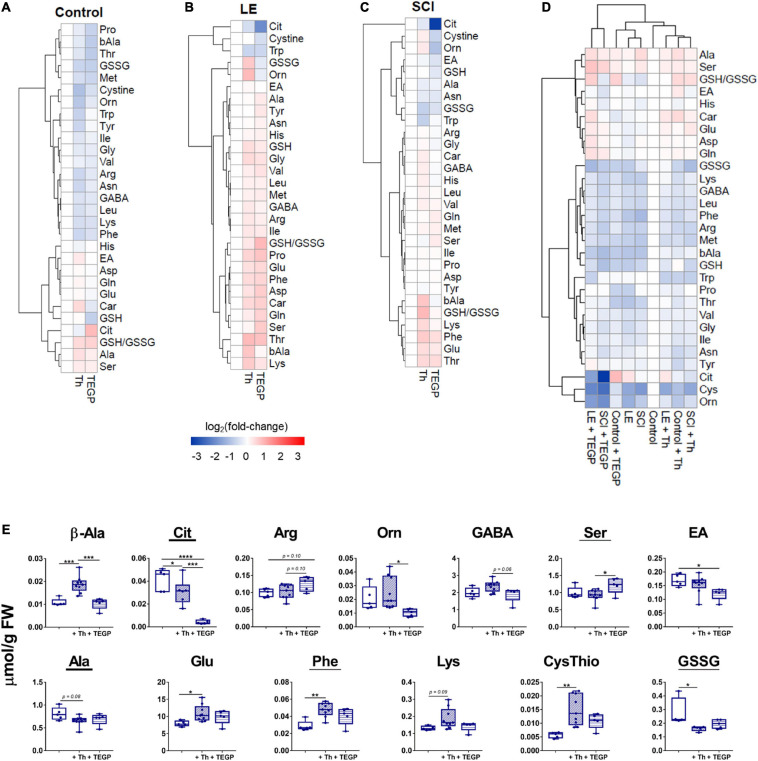
Physiological-state-dependent effects of the OGDHC regulators thiamine and TEGP on the metabolic profiles of cerebral cortex of the control and operated rats. **(A)** Non-operated rats (Control); **(B)** rats subjected to LE; **(C)** rats subjected to SCI; **(D)** all groups; **(E)** metabolic markers, discriminating the action of thiamine and/or TEGP in SCI animals. Statistically significance of the changes is determined by one-way ANOVA followed by *post hoc* Tukey’s test. Underlined are the metabolites changed in SCI vs. LE animals, according to Table 1. Metabolites are abbreviated as in Table 1. Clustering of metabolites and heatmaps in **(A–C)** are created using *ward.D2* and *pheatmap* package in RStudio. The data in **(A–C)** are presented as log_2_ (fold-change), where fold-change is a proportion of the mean metabolite level in the thiamine (Th) or TEGP-treated groups to the corresponding mean in the reference group. The color code of the fold-changes is defined by the scale bar under the heatmaps **(A–C)**. On the graphs, different *p*-values are shown by asterisks as described in Methods under Statistical Analysis. Number of animals in each group: LE, *n* = 5; SCI, *n* = 5; LE + Th, *n* ≥ 6; SCI + Th, *n* ≥ 5, LE + TEGP, *n* = 6; SCI + TEGP, *n* = 5.

The animal-state-dependent action of the OGDHC regulators on the enzymatic network is manifested also in the brain metabolomes. Figure 7 presents the thiamine or TEGP-induced changes in the profiles of the brain amino acids and related compounds, assessed in each of the states, i.e., after the treatments of the control rats (Figure 7A), rats after LE (Figure 7B) or SCI (Figure 7C), or by comparison of all the experimental groups (Figure 7D). The metabolic changes are presented on the cluster maps of Figure 7 as the logarithms of fold-changes in the metabolite levels resulting from the action of a factor. Each column of the map characterizes a specific physiological or pathological state of the brain metabolic networks, corresponding to the action of studied factors (shown below the Figures 7A–D). The changes in different metabolites form the clusters shown as a tree at the left of Figures 7A–D. The level of similarity between different groups of animals is shown by the clusters above Figure 7D. The clustering procedure helps to reveal both the specific and common features of the compared states.

In accordance with the different effects of the TEGP and thiamine treatments on the brain metabolic enzymes in different animal states (Figures 3, 4, 6), the heatmaps reveal the state-specific changes of metabolome after the administration of TEGP or thiamine. Remarkably, in the LE-subjected animals (Figure 7B), the treatments-induced changes generally oppose those in the control group (Figure 7A), while in the SCI-subjected animals the metabolic changes are more complex. The heatmaps presented in Figures 7A–C also reveal some specific markers of the treatments. For example, in the control rats (Figure 7A), an increase in the citrulline level marks the action of TEGP. In contrast, a decrease in citrulline level is a specific marker of the TEGP action in the LE (Figure 7B) and SCI (Figure 7C) animals, with the strongest decrease in the latter case. TEGP and thiamine after LE have opposite effects on the GSSG and ornithine levels (Figure 7B). After SCI, only the ornithine levels discriminate the action of thiamine and TEGP (Figure 7C). Other metabolites in the heatmaps confirm that the most sensitive discriminators of the thiamine and TEGP treatments depend on physiological state. Hence, to find biochemical markers of the positive action of thiamine in the SCI-subjected animals (Figure 5), we searched for the metabolites which exhibit statistically significant changes, or tended to those, in the three groups of SCI animals: the thiamine-treated, TEGP- treated and non-treated ones.

The metabolic markers of the thiamine-treated vs. non-treated SCI animals are glutamate, phenylalanine, lysine, cystathionine, alanine and GSSG (Figure 7E). From those, the levels of glutamate, phenylalanine, lysine and cystathionine correlate positively with the locomotor performance scores (Table 2), while the thiamine-induced decreases in alanine and GSSG point to the optimized TCA flux and redox metabolism. Metabolites showing significant differences between the SCI animals treated with thiamine vs. both the non-treated and TEGP-treated SCI animals, are β-alanine and citrulline. The markers discriminating the actions of TEGP and thiamine in SCI animals, are arginine, ornithine, GABA and serine, with arginine and ethanolamine showing the difference in the TEGP-treated vs. non-treated SCI animals (Figure 7E). Thus, the specific indicators of the TEGP action in the cortices of SCI animals are related to metabolism of NO^⋅^ and urea cycle (citrulline, arginine, ornithine), as well as metabolism of phospholipids (serine and ethanolamine).

Most of the metabolites shown in Figure 7E, change similarly in the LE and SCI animals. The difference between the SCI and LE animals is manifested only in the response of GSSG to the thiamine administration, and in the responses of ethanolamine and GABA to the TEGP administration (Table 1). In the treated LE animals, these indicators do not change (ethanolamine and GABA) or change in the direction opposite to that in the treated SCI animals (GSSG). Thus, GSSG and ethanolamine with GABA demonstrate the responses to thiamine and TEGP, respectively, which are specific for SCI animals and not observed in LE animals.

Overall, the metabolic profiling points to an essential role of the OGDHC-controlled checkpoint in the long-term changes of the redox-related signaling (NO^⋅^, GSSG) and homeostasis of neurotransmitters (glutamate, GABA) and their precursors. This conclusion is further supported by comparison of all the studied metabolic states with the metabolome of the intact rat cortex (Figure 7D). According to the hierarchical clustering procedure, whose results are shown above the metabolic profiles in Figure 7D, the metabolome of TEGP-treated control rats belongs to the sub-cluster including the metabolomes of LE and SCI animals. This finding indicates similarity of the brain metabolomes after either the administration of the OGDHC proinhibitor or the operations (LE and SCI). In other words, short-term inhibition of OGDHC in control animals causes chronic changes in the brain metabolomes, which mimic those induced by the spinal operations (Figure 7D). The observation supports essential role of the OGDHC function in the changes of the amino acid profiles observed in the cortices of the LE or SCI animals. Remarkably, administration of TEGP to the LE and SCI animals causes the separate clustering of metabolomes in such cortices (Figure 7D). This cluster demonstrates the highest, compared to all other states, level of metabolic changes, particularly the highest citrulline decrease in the TEGP-treated SCI animals (Figure 7D).

In contrast to the TEGP treatment, the thiamine-treated rats after LE and SCI are clustered together with the thiamine-treated control rats and non-treated animals (Figure 7D), pointing to the thiamine-dependent normalization of the chronic changes after LE and SCI. The clustering results are in good accord with the specific comparisons of SCI vs. LE animals, presented in Table 1, where TEGP aggravates and thiamine alleviates the difference between the brain metabolic profiles of these two groups. Furthermore, the metabolic similarity of the operated thiamine-treated animals to the control state (Figure 7D) agrees with the positive effect of thiamine on the BBB scores of SCI animals (Figure 5).

### Influence of the OGDHC Regulators on the Expression of Master Metabolic Regulators Sirtuin 5 and p53 in the Cerebral Cortex

OGDHC is the main producer of succinyl-CoA for the protein succinylation (Gibson et al., 2015), representing a way of metabolic regulation where the mitochondrial desuccinylase sirtuin 5 is involved (Kulikova et al., 2018). Sirtuin 5 is neuroprotective under ischemic conditions in the brain (Morris-Blanco et al., 2016), providing for homeostatic rearrangements associated with the chronic inflammation after SCI (Vachharajani and McCall, 2020). Besides, given the rate-limiting role of OGDHC in the TCA cycle (Bunik and Fernie, 2009) and an essential role of the brain GDH in the glutamate flux, the chronic downregulation of both OGDHC and GDH in the brain cortex after SCI (Figure 2) points to significant changes in the mitochondrial energy metabolism. These are known to be controlled by the transcriptional regulator p53, which, in particular, increases after cerebral ischemia, enhancing the neuronal apoptosis (Raz et al., 2011). Hence, expression of sirtuin 5 and p53 has been assessed in the experimental animal groups. Acetylation of p53 has been checked, as thiamine is known to be involved with the protein acetylation (Mkrtchyan et al., 2015; Aleshin et al., 2020).

**FIGURE 8:**
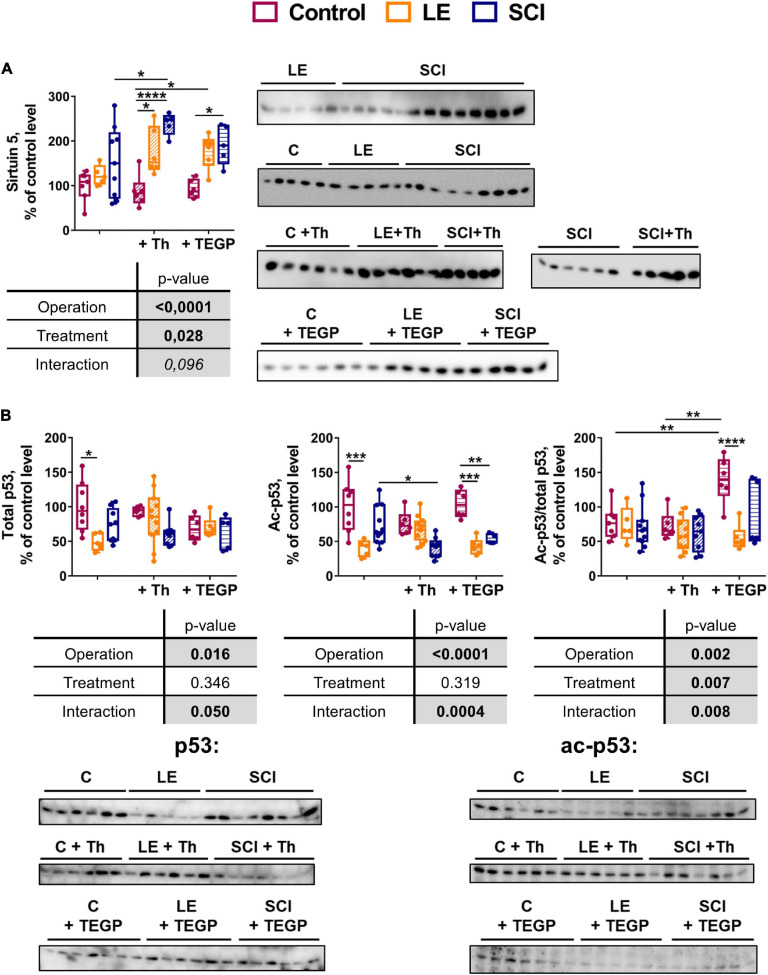
Comparison of the effects of the OGDHC regulators thiamine (Th) and TEGP on the expression of sirtuin 5 **(A)** and total or acetylated p53 **(B)** in cerebral cortex of the experimental rat groups. The expression is assessed by the western-blotting procedures described in Methods. Data for the different animal groups are expressed as percentage of the corresponding values determined in the non-operated (control, C) rats. The two-way ANOVA and *post hoc* Tukey’s test are used to determine statistical significance of differences. On the graphs, different *p*-values are shown by asterisks as described in Methods under Statistical Analysis. In the tables, significances (*p* ≤ 0.05, in bold), according to the two-way ANOVA, are highlighted in gray. Only the statistically significant differences between the pairs of groups varying in a single factor (operation or treatment), are shown.

As shown in Figure 8A, the wide variations in the sirtuin 5 expression in the non-treated SCI animals are not observed in the control and LE animals. Remarkably, after the thiamine treatment, the SCI animals become more uniform in the sirtuin 5 level, showing a significant increase in its level in the brain, compared to the non-operated (control) animals. A similar effect of lower amplitude is observed in the thiamine-treated LE animals. Thus, thiamine administration does not affect the sirtuin 5 level in the control animals, but increases it in the LE and SCI animals (Figure 8A).

Compared to the thiamine administration, that of TEGP affects the sirtuin 5 level in a similar, but less pronounced, way. That is, the sirtuin 5 expression significantly increases in the SCI animals after TEGP administration, with the increase not reaching statistical significance in the LE animals (Figure 8A). Thus, the action of thiamine and TEGP in SCI animals is linked to the sirtuin 5 expression. However, only in the thiamine-treated SCI animals the level of sirtuin 5 becomes significantly higher, compared to the non-treated SCI animals (Figure 8A). Obviously, the thiamine-increased expression of the neuroprotective sirtuin 5 contributes to the positive physiological effect of the thiamine treatment on the locomotor performance after SCI (Figure 5). This is substantiated by a significant positive correlation of the sirtuin 5 protein level with physiological recovery after SCI (Table 2).

In the non-treated animals, both the total and acetylated p53 decrease in the LE group, compared to the control animals (Figure 8B). Thiamine administration attenuates the decreases in p53 and acetyl-p53, which are observed in the non-treated LE animals, compared to the control animals (Figure 8B). In the SCI animals, thiamine administration causes a proportional decrease in the acetylated and total p53. The proportionality is evident by no effect of thiamine on the acetyl-p53/p53 ratio (Figure 8B), although for the acetyl-p53 levels the thiamine-induced decrease reaches statistical significance. In view of no change in the p53 acetylation level, i.e., acetyl-p53/p53 (Figure 8B), a higher statistical significance of the change in acetyl-p53, compared to p53, may be due to a better sensitivity of the acetyl-p53 detection. Accordingly, only the level of acetyl-p53, but not the ratio of acetyl-p53 to p53 (the level of p53 acetylation), shows statistically significant negative correlation with the locomotor recovery (Table 2).

In contrast to the thiamine action on (acetyl)p53, a strong increase in the level of p53 acetylation is observed in the TEGP-treated non-operated animals, compared to both the non-treated and thiamine-treated non-operated animals (Figure 8B). This TEGP-induced increase in the p53 acetylation is attenuated by LE and SCI, manifesting a strong interaction between the TEGP treatment and the operations (Figure 8B). Thus, independent of thiamine administration, the treatment of the control animals with the pro-inhibitor of OGDHC increases the level of p53 acetylation, with the effect not observed in the operated animals.

As a result, thiamine administration does not change the level of p53 acetylation, decreasing the brain level of both the total and acetylated p53 in SCI animals. In contrast, TEGP administration increases acetylation of p53 in non-operated animals (Figure 8B), where it also increases the OGDHC activity (Figure 6B).

## Discussion

### Function of Brain OGDHC, Downregulated by SCI, Is Linked to Expression of Sirtuin 5 and p53

This work has shown that in both the spinal cord and brain, a key mitochondrial multienzyme complex, OGDHC, is downregulated as a chronic consequence of severe SCI, with the downregulation linked to decreased activity of the immediate metabolic partner of OGDHC—GDH. The extension of the spinal trauma consequences to the distant area of CNS should be taken into account in the studies which develop the damage markers of neurotrauma, using non-damaged area of the same organism as a matched control (Bogoslovsky et al., 2018).

Compared to the brain, the spinal cord surrounding the neurotrauma area demonstrates a much more pronounced downregulation of OGDHC, that is accompanied by decreased activities of not only GDH, but also MDH and ME. Thus, the stronger the OGDHC downregulation, the more extensive the changes in the affiliated metabolic pathways. The essential metabolic impact of OGDHC is due to the TCA-cycle-limiting role of this complex, functioning in a highly regulated branch point of metabolic network. The TCA cycle with the downregulated OGDHC produces less NADH for oxidation in the electron transport chain, thus causing an impairment of such a major mitochondrial function as oxidative phosphorylation (Gibson et al., 2005; Bunik and Fernie, 2009). In addition, metabolism of amino acids is affected under these circumstances, according to the role of the TCA cycle and OGDHC in the amino acid biosynthesis and degradation (Santos et al., 2006; Bunik and Fernie, 2009; Graf et al., 2009; Trofimova et al., 2012; Araujo et al., 2013; Graf et al., 2020).

Metabolic significance of the long-term changes in the brain OGDHC function is further supported by the responses of the two master metabolic regulators, sirtuin 5 and p53, to such changes. Our experiments show that the thiamine administration increases the levels of sirtuin 5 under pathological conditions of neurodegeneration (SCI). Similar effect, although to a lower extent, is observed after the treatment of animals with a pro-inhibitor of OGDHC, TEGP. As the OGDHC inhibition is known to induce a compensatory increase in the thiamine influx in the brain (Mkrtchyan et al., 2016), the increased level of intracellular thiamine after the TEGP treatment may underlie the similarity in the thiamine and TEGP action on the sirtuin 5 expression. In contrast, lower intracellular levels of thiamine may cause decreased sirtuin 5 level observed after metformin treatment (Buler et al., 2016), as metformin is known to inhibit intracellular thiamine transport (Aleshin et al., 2019).

Unlike the changes in the sirtuin 5 levels, which are similar in the thiamine or TEGP-treated animals, the changes in the p53 expression and acetylation are different in these animal groups. The thiamine-induced decrease in expression of both p53 and its acetylated form in SCI animals is in good accord with the normalizing action of thiamine on the brain amino acid metabolism and improvement in post-SCI locomotor recovery by thiamine. In contrast, treatment with the pro-inhibitor of OGDHC TEGP increases acetylation of p53 in the brain of non-operated animals, where metabolic profiles approach those induced by LE and SCI. No changes in p53 acetylation in the brains of the TEGP-treated LE and SCI animals suggest decreased homeostatic regulation after the operations, that is also seen in the highest perturbation of the brain metabolic profiles in these animals, compared to other groups studied.

### Metabolic Markers and Physiological Significance of the Long-Term OGDHC Downregulation in the Cerebral Cortex of SCI-Affected Rats

The physiological flux through OGDHC is not equal to the enzyme activity determined *in vitro* under the substrate saturation, and may better be assessed by the relevant metabolic markers. A lower, compared to LE animals, flux through the brain TCA cycle with the SCI-downregulated OGDHC is manifested by the SCI-induced increases in alanine, aspartate and proline. The perturbed brain metabolism of amino acids, observed in our study 8 weeks after SCI, is in good accord with the study of TBI patients who demonstrate changed amino acid profile in the plasma after the rehabilitation (Aquilani et al., 2003). Importantly, the levels of glutamate and GABA do not differ between the LE and SCI groups, suggesting a compensatory response of the metabolic network to stabilize the levels of the major neurotransmitters. The observed changes in the assayed enzymes represent specific components of such a response. Co-regulation of OGDHC with the enzymes metabolizing malate and glutamate, is shown in SCI and LE animals and in our experiments with administration of thiamine and TEGP. Functions of the co-regulated enzymes are known to be modified by post-translational acylations. Efficiency of the OGDHC-dependent succinylation (Gibson et al., 2015) is expected to be enhanced within the OGDHC-including supramolecular structure, regulating the distribution of the substrate flux between the catabolic (energy production) and anabolic (production of biosynthetic intermediates) reactions. The supramolecular complex between mitochondrial MDH and OGDHC is mediated by the mitochondrial aspartate aminotransferase (Fahien et al., 1988). OGDHC directly binds GDH (Fahien et al., 1989), which undergoes a number of regulatory acylations (Bunik et al., 2016). Physiological role of these modifications is shown by our finding that the thiamine-optimized flux of acetyl moieties through the TCA cycle in the SCI animals not only normalizes the brain metabolic profiles, but also increases acetylation of the GDH K503 residue. In good accord with our previous study (Aleshin et al., 2020), the modification activates GDH whose function is positively correlated with the locomotor performance. Thus, competitive posttranslational acylations, such as succinylation and acetylation, of the OGDHC-comprising supramolecular complex may contribute to the coupled changes of the glutamate and malate metabolism, observed in SCI animals.

Our finding of the positive correlation of BBB with the brain desuccinylase sirtuin 5 supports the adaptive nature of the OGDHC downregulation. The adaptation would decrease production of succinyl-CoA for the protein succinylation, which appears to be excessive in the SCI-perturbed brain metabolism requiring up-regulation of sirtuin 5. On the other hand, the TCA-flux-limiting OGDHC is also crucial for *de novo* biosynthesis of glutamate from glucose. Under physiological conditions, a strong positive correlation between the OGDHC activity and glutamate level, which in turn positively correlates with the GABA level, is observed in the rat cerebral cortex, but the correlation decreases under pathological conditions (Mkrtchyan et al., 2018a). In the brain metabolism perturbed by SCI, the downregulation of OGDHC may spare glutamate from the OGDHC-dependent degradation in the TCA cycle. Physiological importance of the brain metabolism of glutamate after SCI is supported by the most significant and strongest correlation of the cortex glutamate level with the BBB score, added by the positive correlation trend of BBB with the glutamate-generated GABA. The trends of positive correlations of BBB score with the branched chain amino acids leucine and isoleucine agree with the known role of these amino acids in supporting both the TCA cycle flux at insufficient OGDHC function (Santos et al., 2006; Trofimova et al., 2012) and the glutamate homeostasis during the neurotransmission (Yudkoff, 2017; Hull et al., 2018).

Earlier works considered negative outcome of the neurotrauma-decreased enzymatic activities for the energy metabolism during a short-term period up to several days after the trauma (Sharma et al., 2009; Mannino et al., 2018; Mkrtchyan et al., 2018b; Lazzarino et al., 2019; Pandya et al., 2019). On the other hand, levels of free amino acids and related peptides, such as glutathione, are known to be of marker significance in different neuropathologies, including not only traumatic injuries of CNS, but also individual action of associated pathogenic factors, such as hypoxia and perturbed neurotransmitter metabolism (Dash et al., 2016; Amorini et al., 2017; Griffin and Bradshaw, 2017; Graf et al., 2020; Rana et al., 2020). To the best of our knowledge, studies of the long-standing consequences of spinal trauma have not assessed the metabolic changes in the brain. Nevertheless, neuroinflammation and reduced neurogenesis in the brain, associated with cognitive impairments, have been shown among the consequences of SCI (Jure and Labombarda, 2017; Kajstura et al., 2018). These phenomena are strongly linked to metabolism, particularly to the regulation of the mitochondrial TCA cycle and associated amino acid metabolism, where OGDHC is a major player (Bunik et al., 2007; Sen et al., 2012; Diaz-Munoz et al., 2015). The long-term (8 weeks after SCI) changes in OGDHC and affiliated enzymes of central metabolism may not only contribute to post-SCI metabolic adaptations, but also perturb immune response (Diaz-Munoz et al., 2015) and be associated with neurodegenerative processes (Gibson et al., 2005; Mkrtchyan et al., 2018b). One may therefore conclude that the OGDHC downregulation as an adaptation to the SCI-induced changes is a double-edged sword.

### Knowledge-Based Insights to Development of Therapeutic Approaches in SCI

Perturbations in oxidative metabolism that belong to primary consequences of traumatic injury, increase the reactive oxygen and nitrogen species. The ensuing oxidative modifications of metabolic enzymes cause a vicious cycle of the damage build up (Bunik et al., 2007). Provision of the damaged tissue with the enzyme ligands, such as coenzymes, substrates or their analogs, shortly after neurotrauma, increases the presence of the enzyme-ligand complexes with the active site residues protected from irreversible modifications. Preventing the primary damaging action of the perturbed oxidative metabolism, the ligand binding would inhibit also the long-term reinforcement of the damage. Such a mechanism is in good accord with our observations that a single administration of the enzyme effectors has long-term consequences. The mechanism also agrees with the dependence of the consequences on the physiological state of the treated animals, as such states differ in the levels of not only normal metabolites, but also damaging species. Xenobiotics like the phosphonate analogs of 2-oxoglutarate, are supposed to be detoxified and/or excreted shortly after their single administration, as most of the water-soluble drugs do. Hence the long-term changes of the brain metabolism, observed after the administration of TEGP, appear to manifest the long-term consequences of the relatively short-term metabolic perturbations. An excess of the water-soluble thiamine is also easily excreted, but first of all the liver, which is the thiamine storage organ, is saturated by the vitamin. Although the thiamine levels in the brain are tightly regulated (Aleshin et al., 2019, 2020), the buffering function of the liver may contribute to the long-term effects of a single high dose of thiamine.

**FIGURE 9:**
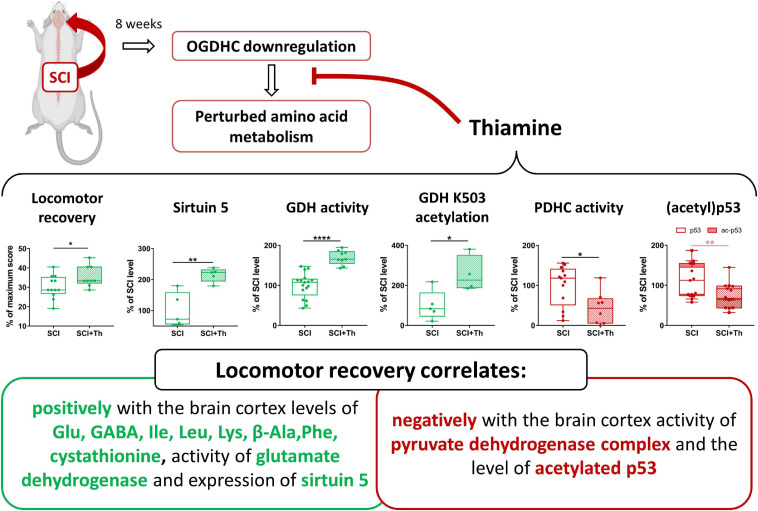
Chronic consequences of SCI and their alleviation by a single post-SCI administration of thiamine. Statistical significance of differences in the brain cortex biochemical parameters of the thiamine-treated and non-treated SCI rats is shown on the graphs, estimated by *t*-test, after the normal data distribution in each group was confirmed using Shapiro-Wilk test. Different *p*-values are shown by asterisks as described in Methods under Statistical Analysis.

Due to the role of OGDHC in the TCA cycle flux and amino acid metabolism, downregulation of OGDHC is supposed to be among the leading causes for development of neurodegenerative diseases (Gibson et al., 2005). In this regard, the pleiotropic metabolic action of thiamine, which also helps the post-SCI locomotor recovery, may be considered as a solution to overcome the negative consequences of the OGDHC downregulation after SCI. Our data about the positive action of the post-SCI administration of thiamine on the parameters correlating with the BBB scores, are summarized in Figure 9. The results show that it is not the total OGDHC activity assayed under saturating concentrations of the OGDHC coenzyme ThDP, that is changed by the thiamine administration. However, thiamine may increase the amplitude of metabolic oscillations supporting neurotransmission, through the oscillating supply of the OGDHC activator ThDP, regulated by cyclic hydrolysis/phosphorylation and/or intracellular transport of thiamine and/or derivatives (Mkrtchyan et al., 2015; Bunik and Aleshin, 2017, and references therein). In view of the regulatory role of the oscillation amplitude in cellular signaling, exemplified by oscillations of another OGDHC activator, Ca^2+^ (Qi et al., 2020), such a mechanism may support the metabolic action of thiamin at a constant expression of the functionally competent OGDHC assayed *in vitro*. This action of thiamine may be undetectable by *in vitro* assays of OGDHC, yet contributing to the observed concerted changes in the OGDHC-affiliated metabolic network and normalization of the amino acid profile, supporting the positive physiological action of thiamine.

One should also consider that the pleiotropic action of thiamine is not necessarily limited to the studied enzymes of the 2-oxoglutarate/glutamate and pyruvate/malate metabolism, but may affect also other thiamine-dependent enzymes and pathways. For instance, involvement in the consequences of SCI of less studied phenomena, such as thiamine metabolism, is suggested by the marker role of the thiamine monophosphatase in the primary afferents sprouting in the spinal cord (Schwegler et al., 1995). In this regard, application of natural metabolic regulators may provide more beneficial combinatorial therapies to fight chronic consequences of neurotrauma, compared to the “one target” metabolic regulators, such as TEGP.

Our observation of positive correlations of post-SCI locomotion recovery, assessed by the BBB score, with β-alanine, phenylalanine, lysine and cystathionine is in good agreement with independent data suggesting nutritional corrections of the neurotrauma-associated consequences. Thus, β-alanine supplementation helps overcoming the post-traumatic stress disorder after TBI (Hoffman et al., 2015, 2017), probably involving the neurotransmitter function of β-alanine (Tiedje et al., 2010). Phenylalanine is among biomarkers discriminating the TBI patients with cognitive disorders from those without such disorders and from healthy controls (Yi et al., 2016). This observation and our own finding of the positive correlation of BBB with the brain phenylalanine levels may be due to the association between the plasma levels of phenylalanine with decreased intracranial pressure and increased oxygenation in TBI patients (Vuille-Dit-Bille et al., 2012). The positive correlation of BBB with lysine agrees with the neuroprotective effect of lysine against ischemia in cerebral cortex (Kondoh et al., 2010). A higher availability of free lysine in the SCI-affected brain may be useful due to excessive modification of the protein lysine residues by endogenous acrolein rising in SCI (Zheng et al., 2013). Besides, free lysine may regulate the NO^⋅^ generation (Boyko et al., 2018) and inhibit the protein lysyl oxidases potentially involved in the neurotrauma-associated modification of extracellular matrix and inflammation (Lin et al., 2020). Lysine also generates polyamines that are part of the stress response systems in living organisms (Olin-Sandoval et al., 2019; Sakamoto et al., 2020; Wu et al., 2020). In this regard, also our observation of the strong positive correlation between BBB and cystathionine deserves attention, because reduced cystathionine is a marker of changed transmethylation and polyamine synthesis, associated with the mTORC1-controlled astrogliosis and pathological anabolism (McKenna et al., 2018). In our model of SCI, such events may well develop due to a long-standing failure to properly re-establish the functions of the damaged spinal cord. Remarkably, other pathways known to be regulated by mTORC1, involve redox homeostasis and glutamine/TCA cycle, shown to be affected by SCI in this and previous (Boyko et al., 2018) studies. Due to the dependence of glutathione biosynthesis on the transsulfurylation pathway generating cysteine from cystathionine (Vitvitsky et al., 2003), the observed cystathionine changes may also be coupled to the changed glutathione metabolism. The identification of these pathways to be affected in the chronic state after SCI suggests that significant role of the OGDHC downregulation by SCI in the perturbed metabolism of amino acids is mediated by mTORC1. Indeed, 2-oxoglutarate is known to stimulate the mTORC1-dependent activation of central carbon metabolism (Suzuki et al., 2018).

## Conclusion

Our study reveals (i) a long-term downregulation of cerebral OGDHC after severe SCI, associated with the enzymatic and metabolomic changes, particularly those of the glutamate and malate metabolism; (ii) positive physiological and biochemical outcome of the post-SCI administration of thiamine, a pleiotropic metabolic regulator and precursor of the OGDHC coenzyme; (iii) mimicking metabolic effects of SCI by administration of the synthetic pro-inhibitor of OGDHC, TEGP, to non-operated animals; and (iv) involvement of (acetyl)p53 and protein deacylase sirtuin 5 in the animal brain response to the OGDHC-directed traumatic interventions and therapeutic treatments. We conclude that pharmacological intervention by a pleiotropic regulator thiamine activates neuroprotective mechanisms, optimizing the function of the OGDHC-dependent metabolic network of the brain after SCI. The resulting alleviation of negative consequences of SCI on the CNS functions is manifested in improved locomotor recovery of animals.

## Data Availability Statement

The original contributions presented in the study are included in the article/Supplementary Material, further inquiries can be directed to the corresponding author/s.

## Ethics Statement

The animal study was reviewed and approved by Bioethics Committees of Russian Cardiology Research-and-Production Complex (Protocol No. 3, 23.03.2016).

## Author Contributions

All authors contributed substantially to the conception, acquisition and analysis of the data and drafting the work, provided final approval of the version to be published, as well as agree to be accountable for all aspects of the work.

## Conflict of Interest

The authors declare that the research was conducted in the absence of any commercial or financial relationships that could be construed as a potential conflict of interest.

## Footnotes

1 https://www.graphpad.com/quickcalcs/randomize1/

2 https://rstudio.com
