# Supplementary material for: Severe Spinal Cord Injury in Rats Induces Chronic Changes in the Spinal Cord and Cerebral Cortex Metabolism, Adjusted by Thiamine That Improves Locomotor Performance
Source: Front Mol Neurosci. 2021 Mar 29;14:620593. doi: 10.3389/fnmol.2021.620593 (PMC8044794; doi:10.3389/fnmol.2021.620593)
Supplement: Supplementary file 1 [file Data_Sheet_1.docx]

Supplementary Material


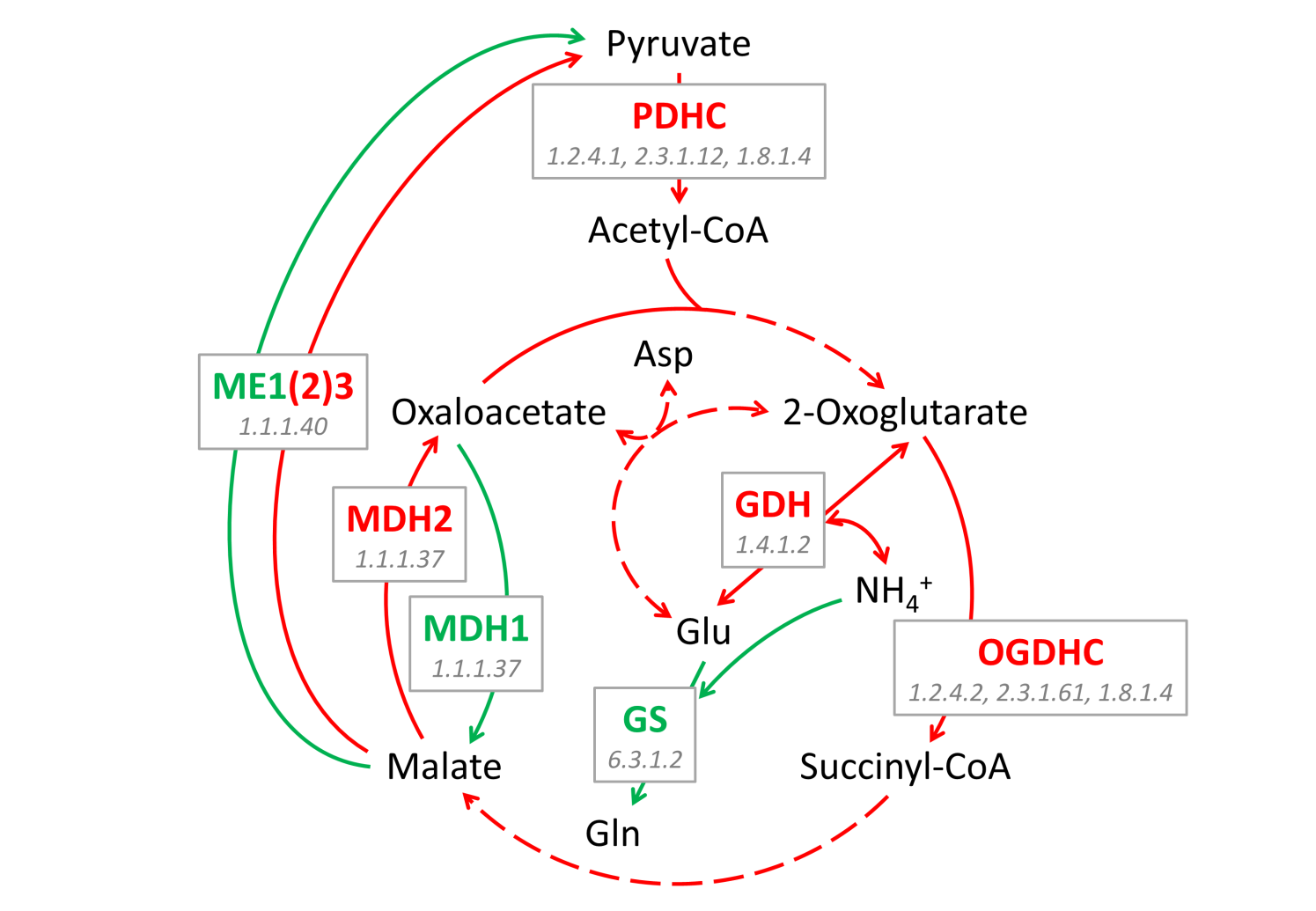


**Supplementary Figure S1. Functional interconnections between the studied enzymes.** The central metabolic flux via TCA cycle and the affiliated reactions involving pyruvate, glutamate and malate, are depicted. Standard three-letter abbreviations are used for amino acids. Cytosolic and mitochondrial reactions are shown in green and red, respectively. The reactions of the enzymes, whose activities are measured in this study, are shown by solid arrows. The dashes arrows correspond to the residual part of the TCA cycle. The assayed thiamine diphosphate-dependent systems are 2-oxoglutarate dehydrogenase complex (OGDHC) and pyruvate dehydrogenase complex (PDHC), each comprising the three catalytic components. For each enzyme the corresponding EC number is provided in grey. The other assayed activities belong to both the cytosolic (MDH1) and mitochondrial (MDH2) isoenzymes of malate dehydrogenase (MDH, EC 1.1.1.37), glutamate dehydrogenase (GDH, EC 1.4.1.2), cytosolic glutamine synthase (GS, EC 6.3.1.2) and the three brain isoenzymes of malic enzyme (ME, EC 1.1.1.40), i.e. the NADP^+^-dependent ME1 and ME3 and NAD^+^-preferring ME2. ME2 is also able to use NADP^+^, but represents a minor isoenzyme in the brain.

**
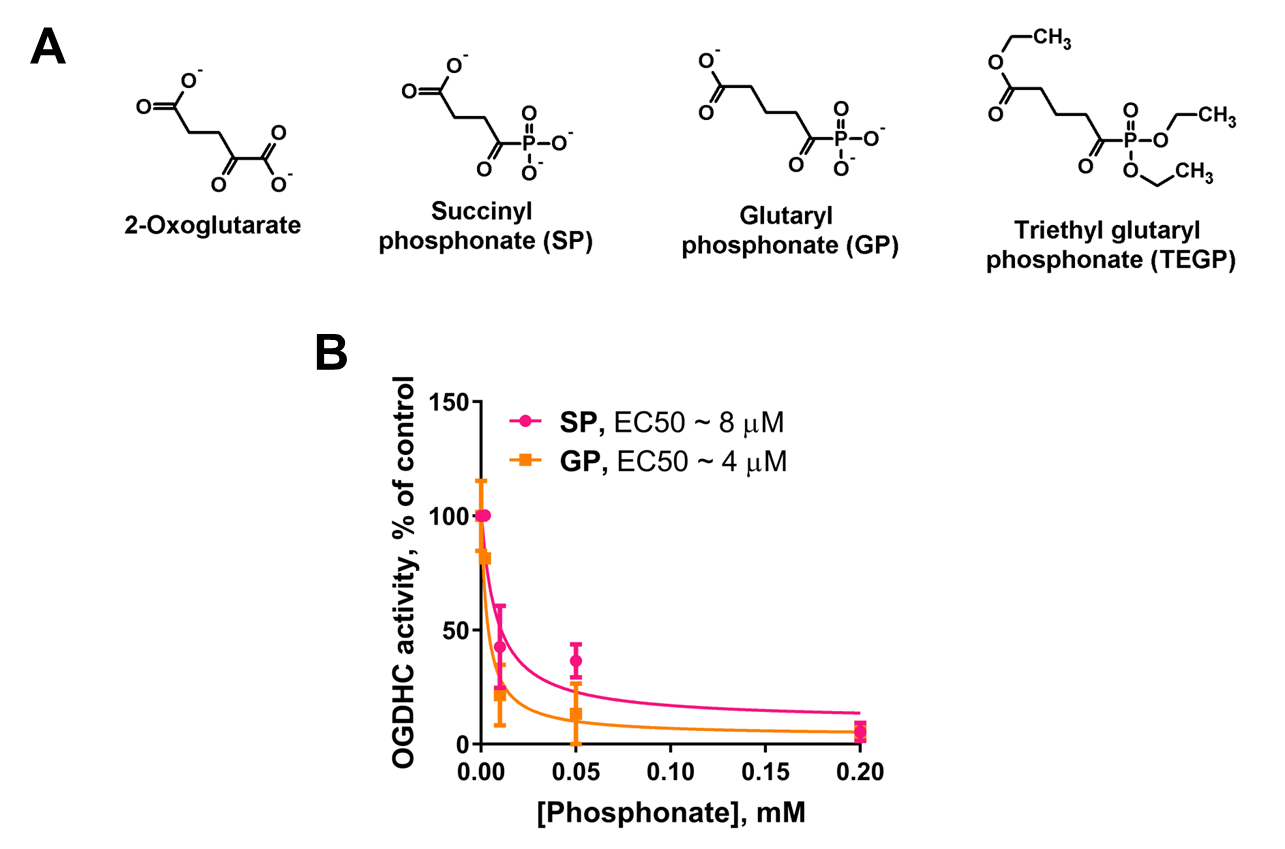
**

**Supplementary Figure S2. Inhibition of the rat brain OGDHC by synthetic phosphonate analogs of 2-oxoglutarate. (A)** Structures of the OGDHC substrate 2-oxoglutarate and its synthetic analogs: succinyl phosphonate (SP), glutaryl phosphonate (GP), and a membrane-permeable precursor of GP, triethyl ester of glutaryl phosphonate (TEGP). **(B)** Comparison of the inhibition of the rat brain OGDHC by GP and SP. The inhibition was assayed at 2 mM concentration of 2-oxoglutarate after a 5 min preincubation with the inhibitors. Experimental data were fitted with Hill equation, with estimated EC50 given on the graph.
